# Urinary Catheterization in Infants: When It’s Knot so Simple

**DOI:** 10.5811/cpcem.2017.11.36438

**Published:** 2018-01-11

**Authors:** David C. Sheridan, Beech Burns, Megan Mickley

**Affiliations:** * Oregon Health & Science University, Department of Emergency Medicine, Portland, Oregon; † Oregon Health & Science University, Department of Pediatrics, Portland, Oregon

## Abstract

Pediatric fever is one of the most common presenting complaints to emergency departments (ED). While often due to a viral illness, in young children without a source the most common bacterial infection is pyelonephritis. For this reason, when no focal source can be identified a urinary specimen is recommended. In young children who are unable to urinate on demand, a straight catheter is required to obtain a sterile specimen. This is generally a benign procedure and is performed frequently in EDs. We report a case of a young girl who underwent straight bladder catheterization and was subsequently found to have a retained catheter that had become knotted in the bladder. This case report highlights a rare complication of this common procedure and describes the technique required to remove the catheter. An understanding of these issues may avoid the need for transfer to a pediatric facility or for subspecialty consultation.

## INTRODUCTION

Children visit emergency departments (ED) often, with over 23 million encounters annually in the United States.^1^ Fever is one of the most common chief complaints for such visits in young children. In the modern era of widespread immunization, occult bacteremia is virtually nonexistent in the well-appearing child; pyelonephritis, however, remains common, accounting for 3–8% of febrile children.^2^,^3^ The American Academy of Pediatrics recommends straight catheter urine samples in young children with risk factors, including female sex, persistent fever above 102º Fahrenheit, and absence of an alternative etiology on exam.^4^ This results in a large number of straight catheterization procedures being performed in EDs across the country, as missing this infection can cause chronic problems from renal scarring.

Pediatric straight catheterization is a relatively fast procedure with a low complication rate of infection or pain and high parental acceptance.^5^ There are few descriptions in the literature regarding complications of this common procedure. This case report describes one potential complication, and the procedure providers can use to correct it.

## CASE REPORT

A six-month-old previously healthy female presented to our tertiary care pediatric ED with fever and vomiting. Her parents noted that she had recently had mild cough and congestion but otherwise no significant preceding symptoms. Over the prior 48 hours, however, she had developed persistent fever with a maximum temperature of 39º Celsius and had been vomiting for the preceding 24 hours. The patient was up to date on vaccines, the product of an uncomplicated full-term pregnancy, and had no significant past medical or family history. Her initial vital signs were remarkable for a heart rate of 185 beats per minute, temperature of 38.7ºC, and normal oxygen saturation. She was evaluated by a board-certified pediatric emergency physician. The exam was notable only for minimal oropharyngeal erythema and a very mild diaper rash without skin desquamation. Due to the constellation of symptoms, physical exam and duration of high fever, a straight catheter urine sample was ordered to evaluate for pyelonephritis.

The attending physician was later called to the room by the ED nurse due to difficulty removing the catheter from the urethra following urine sample collection with a 5F straight catheter. The experienced nurse reported no resistance or difficulty with insertion and immediate drainage of urine. The physician evaluated the patient and noted the catheter to be appropriately exiting the urethral orifice. There was no external evidence of trauma or genitourinary abnormality. Using sterile technique, the catheter was easily advanced forward but, with gentle traction, could not be withdrawn from the urethra.

A single view abdominal radiograph was then obtained that demonstrated a urinary catheter with the distal tip knotted on itself (Image). Pediatric urology consult recommended repeat gentle traction to remove the catheter; this was re-attempted and again was unsuccessful. Pediatric urology subsequently evaluated the patient in the pediatric ED. Their team was also unsuccessful in removing the catheter with traction only. A range of guidewires was available at the bedside for the urology team to attempt to relieve the knot. They inserted a 0.025-inch diameter guidewire into the catheter and advanced it with minimal resistance. This simple procedure was done without any imaging guidance. The guidewire and catheter were then easily removed without a persistent knot. The patient tolerated the procedure well and was able to feed comfortably within 10 minutes. The urine studies returned and were negative for any sign of infection. After tolerating oral feeds and returning to her baseline level of comfort, the patient was discharged with a short course of cephalexin due to the straight catheter manipulation of the sterile GU system. To our knowledge the patient did well and did not require further interventions following discharge.

## DISCUSSION

Few reports in the literature document urethral catheter knotting in the pediatric population. Given that straight catheterization is an extremely common procedure in children, provider knowledge regarding this complication and its potential solutions is critical. Increased awareness may prevent unnecessary subspecialty consultation if the physician feels comfortable advancing a guidewire or may, minimally, allow prompt recognition and urgent transfer to higher levels of care if the knotting occurs in a setting where circumstances do not permit procedural intervention.

CPC-EM Capsule

What do we already know about this clinical entity?

Straight catheters in the pediatric population are common in the emergency department setting, with urinary tract infections being one of the most common bacterial infections.

What makes this presentation of disease reportable?

With the straight catheter procedure so common, complications may arise. Feeling comfortable to deal with this complication is of utmost importance.

What is the major learning point?

If a straight catheter cannot be retracted, one must suspect a knot. Gentle traction is generally successful, but if it isn’t successful then passing a guidewire can untie the knot and allow retraction.

How might this improve emergency medicine practice?

This procedure allows treatment at the bedside directly, eliminating unnecessary transfers or prolonged pain for patients.

There is one previous report of a pediatric emergency patient who experienced a urethral catheter knot that required only gentle traction for removal.^6^ In the case we describe, both the emergency physician and the urologist were unsuccessful in their attempts using that method. The novel feature of our report was the addition of a bedside guidewire procedure to facilitate catheter removal. By using a guidewire that is more firm than the floppy catheter it provides a skeleton to straighten the catheter. By advancing the guidewire slowly, it has the ability to unwind the knot at the end of the catheter. Of note, the procedure required trialing several guidewires of different sizes to assess which could thread easily through the catheter; thus, having a range of guidewire diameters available is essential. As support for this approach, we note that this technique has been used successfully in the radiology suite under fluoroscopic guidance in voiding cystourethrograms.^7^

Though we used radiography to better define this complication, there is one study demonstrating that ultrasound can be used to identify catheter knotting.^8^ Another study used simulation to evaluate risk factors for catheter knotting.^9^ The authors found that catheter size less than 10F – often the size used in pediatric patients such as the one in this report – was a risk factor. Finally, knotting has also been described with indwelling Foley catheters. However, this may be due to the presence of the retention balloon, a component not present in the straight catheters used for urinary sampling.^8^

## CONCLUSION

Urinary straight-catheter sampling complicated by knotting is an infrequent complication and should not deter the provider from appropriate collection of urine in the right clinical scenario. However, knowledge of the different methods used for catheter removal in the setting of this complication is important for any physician treating children. Based on this case report, we recommend gentle traction as first-line therapy and simple guidewire insertion to un-knot the catheter as a second-line procedure. An understanding of these issues may avoid the need for transfer to a pediatric facility or for subspecialty consultation. In addition, it provides a solution to a complication from a common procedure.

## Figures and Tables

**Image:**
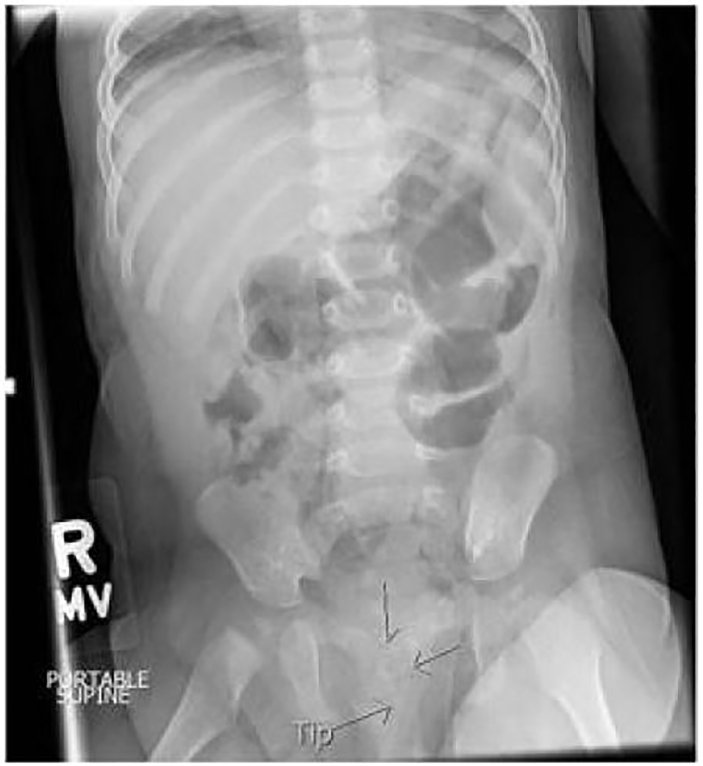
Anteroposterior abdominal radiograph (arrows delineate the catheter location with knot)
